# A clinically feasible circulating tumor cell sorting system for monitoring the progression of advanced hepatocellular carcinoma

**DOI:** 10.1186/s12951-023-01783-9

**Published:** 2023-01-21

**Authors:** Xiu-Yan Huang, Feng Li, Ting-Ting Li, Jun-Tao Zhang, Xiang-Jun Shi, Xin-Yu Huang, Jian Zhou, Zhao-You Tang, Zi-Li Huang

**Affiliations:** 1 Department of General Surgery, Shanghai Jiao Tong University Affiliated Sixth People’s Hospital, 600 Yishan Road, Shanghai, 200233 People’s Republic of China; 2 School of Materials of Science and Engineering, Shanghai Jiao Tong University, 800 Dongchuan Road, Shanghai, 200240 People’s Republic of China; 3 Department of Infectious Disease, Shanghai Jiao Tong University Affiliated Sixth People’s Hospital, 600 Yishan Road, Shanghai, 200233 People’s Republic of China; 4 Institute of Microsurgery on Extremities, Shanghai Jiao Tong University Affiliated Sixth People’s Hospital, 600 Yishan Road, Shanghai, 200233 People’s Republic of China; 5 Liver Cancer Institute and Zhongshan Hospital, Fudan University, 180 Fenglin Road, Shanghai, 200032 People’s Republic of China; 6 Department of Radiology, Xuhui District Central Hospital of Zhongshan Hospital, Fudan University, 966 Huaihai Middle Road, Shanghai, 200031 People’s Republic of China

**Keywords:** Hepatocellular carcinoma, Circulating tumor cells, Lipid magnetic spheres, Immunotherapy, Metastasis

## Abstract

**Background:**

Hematogenous metastasis is essential for the progression of advanced hepatocellular carcinoma (HCC) and can occur even after patients receive multidisciplinary therapies, including immunotherapy and hepatectomy; circulating tumor cells (CTCs) are one of the dominant components of the metastatic cascade. However, the CTC capture efficiency for HCC is low due to the low sensitivity of the detection method. In this study, epithelial cell adhesion molecule (EpCAM)/vimentin/Glypican-3 (GPC3) antibody-modified lipid magnetic spheres (LMS) were used to capture tumor cells with epithelial phenotype, mesenchymal phenotype and GPC3 phenotype, respectively, in order to capture more CTCs with a more comprehensive phenotype for monitoring tumor metastasis.

**Results:**

The novel CTC detection system of Ep-LMS/Vi-LMS/GPC3-LMS was characterized by low toxicity, strong specificity (96.94%), high sensitivity (98.12%) and high capture efficiency (98.64%) in vitro. A sudden increase in CTC counts accompanied by the occurrence of lung metastasis was found in vivo, which was further validated by a clinical study. During follow-up, the rapid increase in CTCs predicted tumor progression in HCC patients. Additionally, genetic testing results showed common genetic alterations in primary tumors, CTCs and metastatic tissues. The proportion of patients predicted to benefit from immunotherapy with the CTC detection method was higher than that for the tissue detection method (76.47% vs. 41.18%, *P* = 0.037), guiding the application of clinical individualized therapy.

**Conclusions:**

The Ep-LMS/Vi-LMS/GPC3-LMS sequential CTC capture system is convenient and feasible for the clinical prediction of HCC progression. CTCs captured by this system could be used as a suitable alternative to HCC tissue detection in guiding immunotherapy, supporting the clinical application of CTC liquid biopsy.

**Supplementary Information:**

The online version contains supplementary material available at 10.1186/s12951-023-01783-9.

## Background

Hepatocellular carcinoma (HCC), one of the most common malignant tumors, has the sixth-highest incidence of all cancers worldwide. It is also the third leading cause of cancer-related death [1]. HCC is highly invasive and metastatic, and these features can readily facilitate distant vascular damage and diffusion [2, 3]. Surgical resection is one of the most effective strategies for HCC; however, even after radical resection, the 5-year cumulative recurrence rate is still 50–70% [4, 5], representing the poor long-term prognosis of patients. Therefore, monitoring and sensing subtle changes in the rates of HCC metastasis and recurrence is critical. However, the present diagnostic sensitivity of imaging and serum tumor biomarkers such as alpha-fetoprotein (AFP) is limited and cannot predict the potential for tumor metastasis [6, 7]. In addition, conventional diagnostic methods have limitations in timeliness, specificity, and sampling. Therefore, there is an urgent need to explore reliable markers for efficacy evaluation and metastasis monitoring in HCC.

Among many cell and molecular markers, circulating tumor cells (CTCs) are more reliable due to noninvasive sampling, real-time information representation, and dynamic reflection of the tumor gene spectrum. CTCs are tumor cells that fall off from tumor lesions into the peripheral blood circulation and play an important role during tumor metastasis [8–11]. Compared with invasive tissue biopsy, CTC detection is easier (so more samples can be collected) and can provides more dynamic information. CTC counting, molecular typing, and downstream gene detection and analysis have broad application prospects in efficacy evaluation, prognosis evaluation, and adjuvant treatment decision-making for patients with tumors [10, 12–14]. Recently, researchers have found that tumor cells undergo epithelial-mesenchymal transition (EMT) when they enter the peripheral blood circulation. Malignant epithelial neoplasms can take advantage of this feature to spread and metastasize [15, 16]. It has been demonstrated that EMT is a process by which epithelial cells switch to the mesenchymal phenotype and increase their expression of mesenchymal markers (vimentin, N-cadherin, fibronectin) while losing their expression of epithelial markers (epithelial cell adhesion molecule (EpCAM), E-cadherin) [17–19].

In previous studies on CTC separation, EpCAM and vimentin were often used as capture targets [20]. However, some CTCs have neither epithelial nor mesenchymal phenotypes in HCC; therefore, CTC sorting with a single target is not sufficient. Glypican-3 (GPC3), a proteoglycan specifically expressed on the surface of the HCC cell membrane, is a clinical biomarker for the diagnosis and targeted treatment of HCC [21, 22]. In addition, enumeration and molecular analysis of CTCs have been highlighted as strategies for evaluating prognosis for cancer management and have emerged as new tools for patient stratification to increase the immunotherapy response rate. Therefore, this study focuses on the innovation of materials application methodology, and focuses on the combined application value of EpCAM-modified LMS (Ep-LMS)/vimentin-modified LMS (Vi-LMS)/GPC3-modified LMS (GPC3-LMS), a novel clinically feasible combined detection strategy was developed to monitor HCC progression and guide immunotherapy application.

## Results

### Characterization of magnetic spheres

**Fig. 1:**
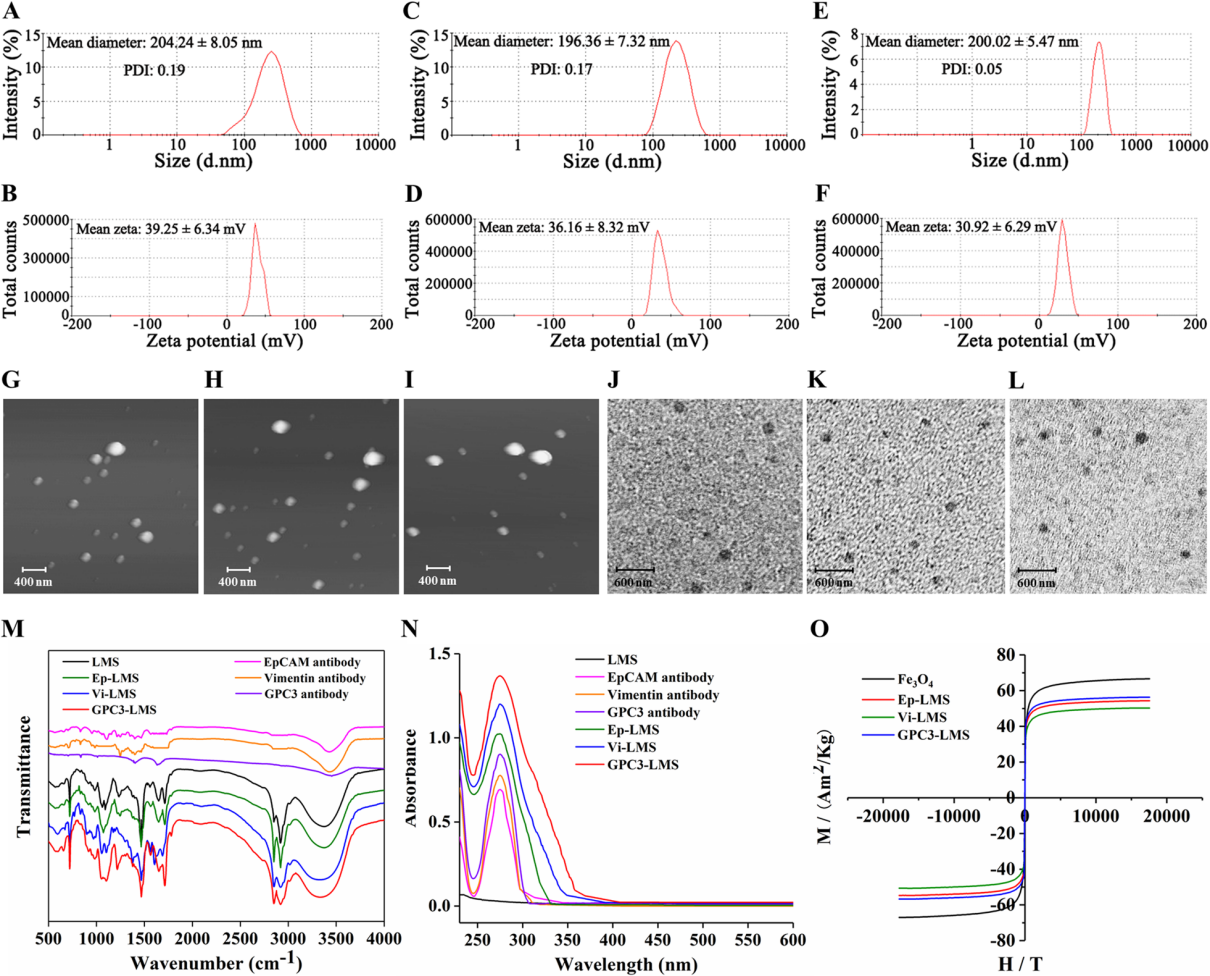
Characterization of magnetic spheres. **A** Particle size distribution of Ep-LMS. **B** Potential distribution of Ep-LMS. **C** Particle size distribution of Vi-LMS. **D** Potential distribution of Vi-LMS. **E** Particle size distribution of GPC3-LMS. **F** Potential distribution of GPC3-LMS. **G** AFM image of Ep-LMS. **H** AFM image of Vi-LMS. **I** AFM image of GPC3-LMS. **J** TEM image of Ep-LMS. **K** TEM image of Vi-LMS. **L** TEM image of GPC3-LMS. **M** IR transmission results spectra. **N** UV transmission results spectra. **O** Saturation magnetization curves

The basic principle of the immune lipid magnetic sphere method is that the antibody with the magnetic sphere binds to the CTC surface antigen, and then CTCs are separated from blood under the action of a magnetic field. Since the existing CTC sorting methods based on the epithelium or EMT are far from sufficient, a novel CTC sorting method in combination with high GPC3 expression for capturing multiple subsets of CTCs is needed. In this study, EpCAM/vimentin/GPC3 antibody-modified LMS were used to capture tumor cells with epithelial phenotype, mesenchymal phenotype and GPC3 phenotype, respectively, a clinically feasible CTC sorting scheme of LMS coupled with different antibodies (anti-EpCAM antibody, anti-vimentin antibody and anti-GPC3 antibody) was designed and constructed to monitor HCC progression for the first time (Additional file 1: Fig. S1). The characterization of Ep-LMS, Vi-LMS and GPC3-LMS is shown in Fig. 1. The average particle size and polydispersity index (PDI) were 204.24 ± 8.05 nm and 0.19 for Ep-LMS (Fig. 1A), 196.36 ± 7.32 nm and 0.17 for Vi-LMS (Fig. 1C), and 200.02 ± 5.47 nm and 0.05 for GPC3-LMS (Fig. 1E), respectively. The zeta potential results of Ep-LMS, Vi-LMS and GPC3-LMS were + 39.25 ± 6.34 mV (Fig. 1B), + 36.16 ± 8.32 mV (Fig. 1D), and + 30.92 ± 6.29 mV (Fig. 1F), respectively. The results of the atomic force microscope (AFM) observation are displayed in Fig. 1G–I. Ep-LMS, Vi-LMS and GPC3-LMS were spherical in different sizes, with regular shapes and no agglomeration. The transmission electron microscope (TEM) observation results (Fig. 1J–L) showed that Ep-LMS, Vi-LMS and GPC3-LMS were round with different sizes, with diameters ranging from 200 to 300 nm. The Fourier transform infrared (FT-IR) spectrum is shown in Fig. 1M. In the FT-IR spectra of LMS, Ep-LMS, Vi-LMS and GPC3-LMS, the infrared absorption vibration peak at 1714.1 cm^−1^ has the characteristic absorption peak of the C = O stretching vibration in the ester group, and 1112.7 cm^−1^ is the stretching vibration absorption peak of C–O–C in the ester bond. 2849.55 cm^−1^ was the stretching vibration absorption peak of -CH-, and 1652.8 cm^−1^ was the stretching vibration absorption peak of –CO–NH– in the amide bond, but the EpCAM, vimentin and GPC3 antibodies did not have these absorption peaks. The UV test results of Ep-LMS, Vi-LMS and GPC3-LMS are shown in Fig. 1N. The absorption peaks of Ep-LMS, Vi-LMS and GPC3-LMS appeared at 260–280 nm. Figure 1O presents the hysteresis curve. The saturation magnetization values for Ep-LMS, Vi-LMS and GPC3-LMS were 54 Am^2^/kg, 25 Am^2^/kg and 35 Am^2^/kg, respectively, which were lower than that for Fe_3_O_4_ (66.5 Am^2^/kg).

### Antibody-modified LMS identifies tumor cells without obvious cytotoxicity

The cytotoxicity of Ep-LMS, Vi-LMS and GPC3-LMS to HCC cells and HUVECs was measured by MTT assay (Additional file 2: Fig. S2). The results showed that the cell survival rate decreased gradually with a gradual increase in the magnetic sphere concentration, and the safe concentration was 50 μg/mL (the cell survival rate exceeded 90% under this concentration). The safe magnetic sphere concentration was calculated to guarantee that CTC detection and application were not hindered by toxicity.

**Fig. 2:**
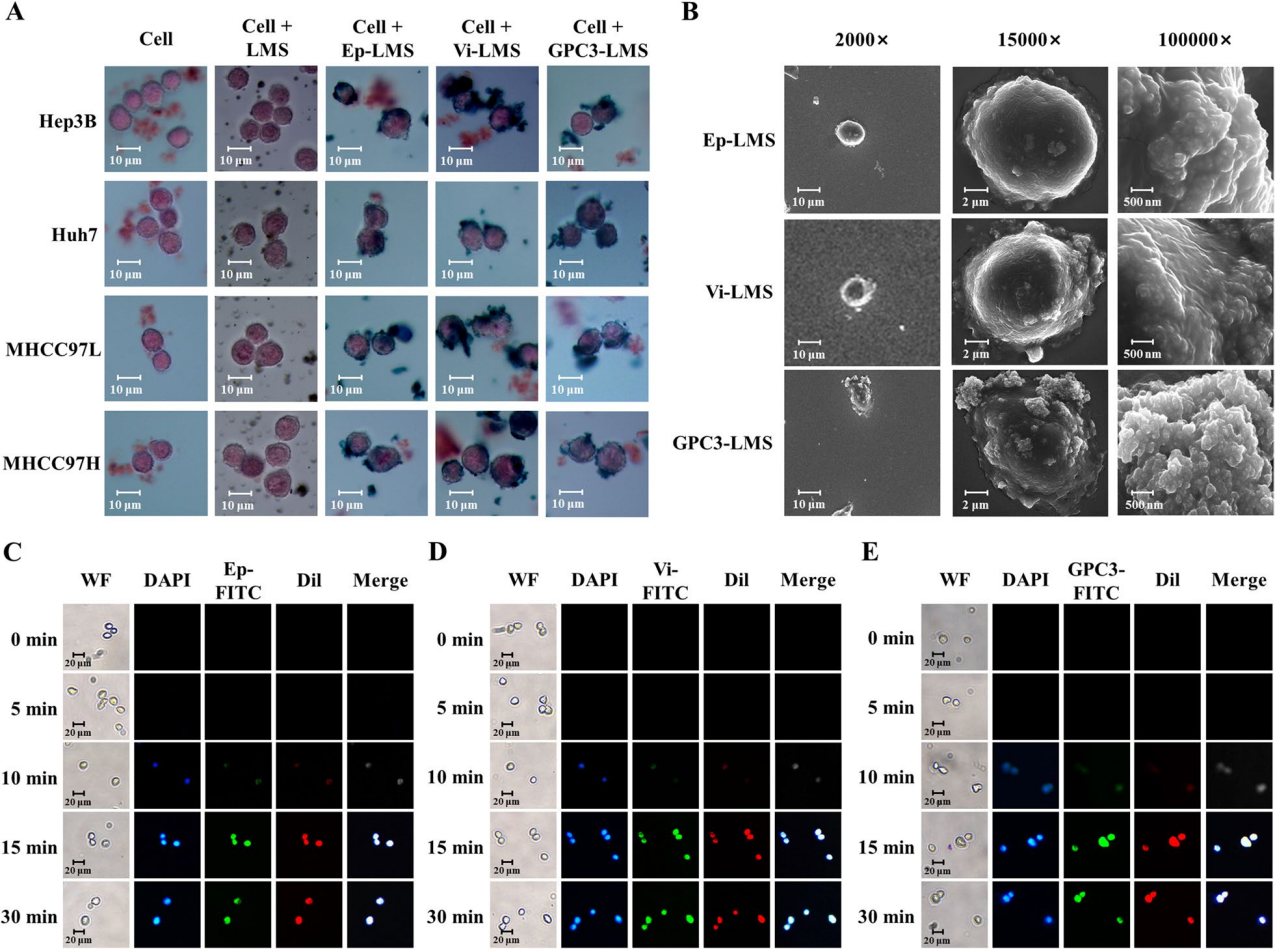
Magnetic sphere distribution and uptake time. **A** Prussian blue staining. **B** SEM observation. **C**–**E** Uptake times of Ep-LMS-FITC, Vi-LMS-FITC and GPC3-LMS-FITC

Prussian blue staining (Fig. 2A) demonstrated that all 4 cell types developed well and had regular shapes. LMS without antibody modification could not recognize cells and spread unevenly on cell surfaces. Ep-LMS, Vi-LMS and GPC3-LMS could target and identify tumor cells and were distributed mainly on the cell surface. Scanning electron microscope (SEM) (Fig. 2B) showed that Ep-LMS, Vi-LMS, and GPC3-LMS were much smaller than cells. Under the field of vision (2000×), the magnetic spheres around cells were concentrated on the cell surface, and only a few magnetic spheres were freely distributed. Under the field of vision (15,000×), a large number of magnetic spheres adsorbed on the cell surface were found, and the aggregated magnetic spheres were enough to isolate the cells by magnetic power. Under the field of vision (100,000×), it could be clearly seen that the particle size distribution of magnetic spheres was between 200 and 300 nm, which is consistent with the results of magnetic sphere characterization. The FITC fluorescence signal in cells gradually increased with time, indicating that the number of LMSs on the cell surface gradually increased with time (Fig. 2C–E), and the optimal uptake time was 15 min (Additional file 3: Fig. S3).

### The combination of Ep-LMS/Vi-LMS/GPC3-LMS improves the capture efficiency in vitro

**Fig. 3:**
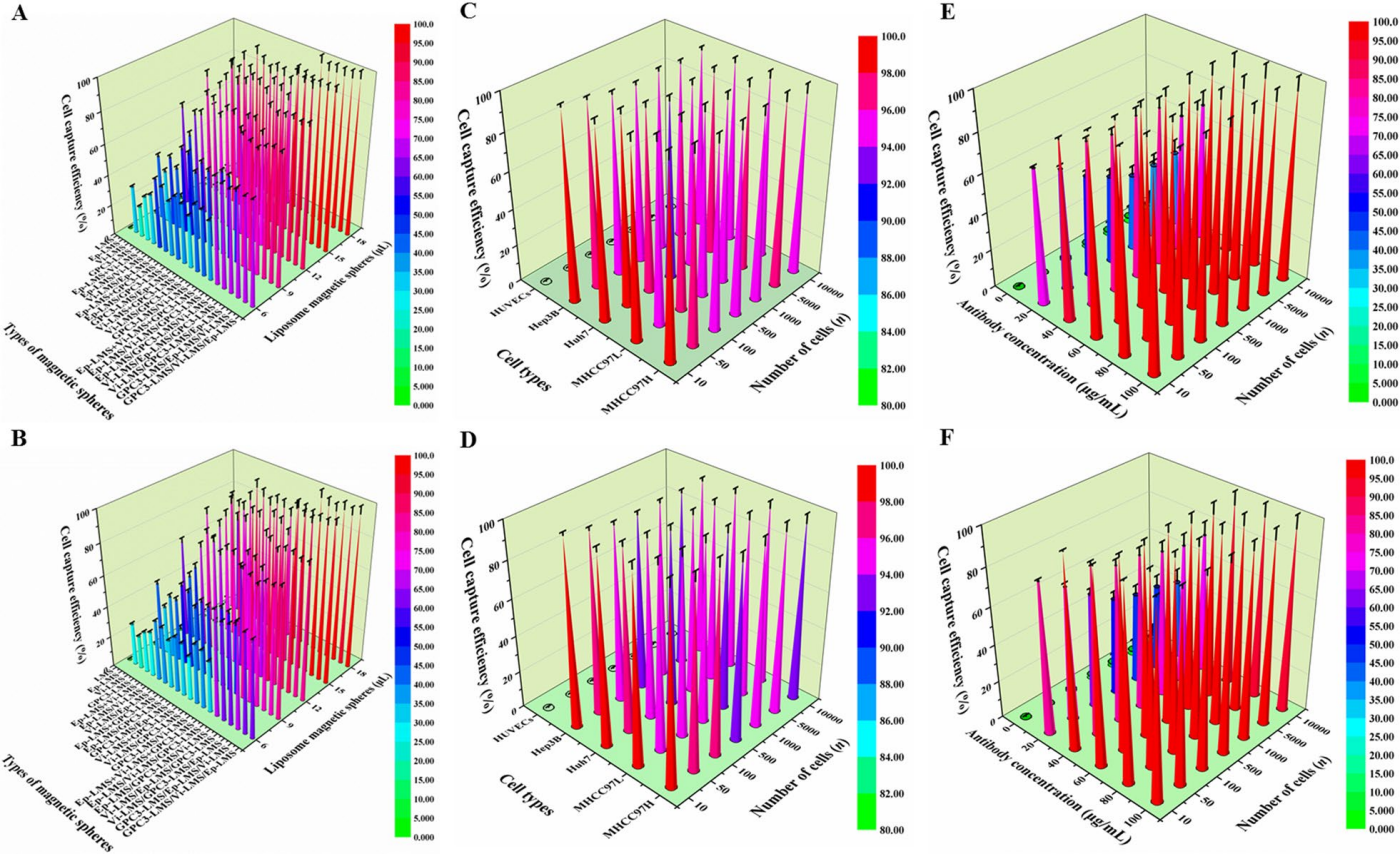
Capture efficiency of Ep-LMS, Vi-LMS and GPC3-LMS. **A**, **B** Capture efficiency of various capture schemes and magnetic spheres for Huh7 cells in the PBS system and simulated blood, respectively. **C**, **D** Sensitivity of the sorting system for 4 HCC cell lines in the PBS system and simulated blood. **E**, **F** Capture efficiency of Huh7 cells by adding magnetic spheres with various antibodies into the PBS system and simulated blood

The cell capture efficiency is shown in Fig. 3. Adding 5 μL (10.8 μg) Ep-LMS/Vi-LMS/GPC3-LMS successively was the best capture scheme (the combination scheme) with a capture efficiency of 98.64% (Fig. 3A), which was verified in the simulated blood. Although the simulated blood was somewhat viscous, the capture efficiency was 97.17% (Fig. 3B). Therefore, Ep-LMS/Vi-LMS/GPC3-LMS was used to detect and analyze the capture efficiency of HUVECs, Hep3B, Huh7, MHCC97L and MHCC97H cells in PBS. The combination scheme demonstrated no significant variations in the capture efficiency of 4 HCC cell lines (*P* > 0.05), with a sensitivity of 98.12% (Fig. 3C) and specificity of 96.94% (Fig. 3D). Cell gradient experiments with varied antibody concentrations showed that the capture efficiency of the system reached the highest when the antibody concentration was 60 µg/mL in both the PBS system and the simulated blood system, and the antibody coupling rate was 2.78% (Fig. 3E, F).

### In vivo models confirmed the feasibility and capture efficiency of the combination sequential capture scheme

**Fig. 4:**
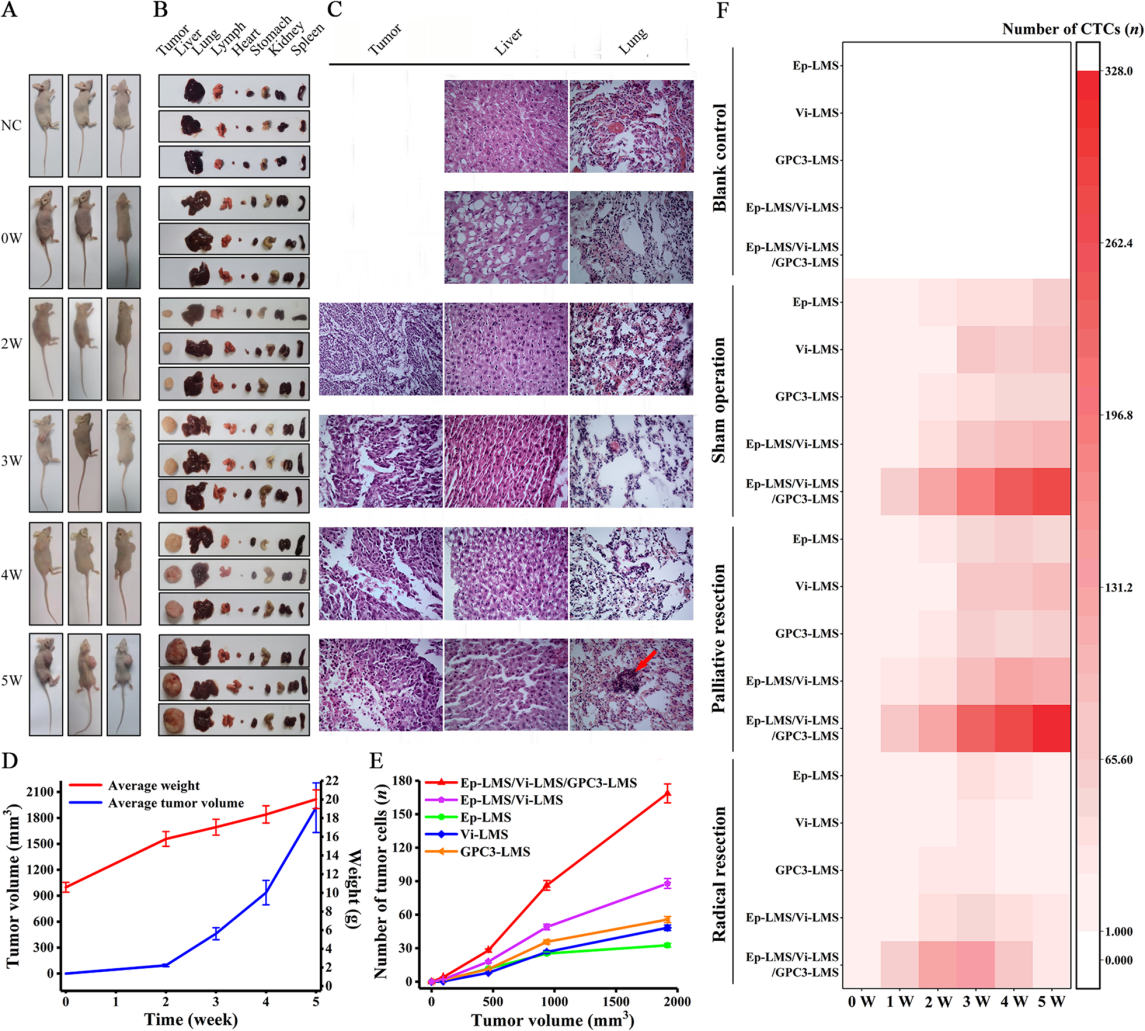
Animal experiments. **A** Nude mice bearing tumors. **B** Tumors and various organs. **C** HE staining. **D** Body weight and tumor volume. **E** Relationship between tumor volume and CTC counts. **F** Application of CTC sorting system in the orthotopic implantation tumor model of nude mice with various types of hepatectomy

HCC tumors and various organs from nude mice are shown in Fig. 4A, B. Pulmonary metastasis occurred at the end of the 5th week after MHCC97H cell implantation subcutaneously in nude mice (Fig. 4C, Additional file 4: Fig. S4). The weights of nude mice increased gradually and evenly, while the tumor volume increased exponentially (Fig. 4D). CTCs captured by Ep-LMS, Vi-LMS and GPC3-LMS were individually identified (Additional file 5: Fig. S5). The number of CTCs gradually increased, but the changing trend was not obvious with tumor volume (Fig. 4E). However, the number of CTCs captured by the two magnetic spheres of Ep-LMS/Vi-LMS, Ep-LMS/GPC3-LMS and Vi-LMS/GPC3 in sequence showed a slight changing trend (Fig. 4E, Additional file 6: Fig. S6). Using the Ep-LMS/Vi-LMS/GPC3-LMS sequential capture system (the combination scheme), we found that a large number of CTC subpopulations were captured at the end of the 5th week (Fig. 4E). The high CTC count indicates that a substantial number of tumor cells accumulated in the circulation and produced lung metastasis, as confirmed by hematoxylin and eosin (HE) staining (Fig. 4C).

Moreover, an orthotopic transplantation tumor model was established to simulate various types of hepatectomy to further verify the clinical feasibility of this combination scheme. The findings revealed that the CTC count was lower in the palliative resection group (PR group), but there was no significant difference between the sham operation group (SO group) and PR group at the same time points after surgery. This may be due to residual lesions after tumor debarking, which kept the number of CTCs high (Fig. 4F). When the combination scheme of Ep-LMS/Vi-LMS/GPC3-LMS was sequentially applied to capture CTCs in the radical resection group (RR group), it was found that the CTC count was significantly reduced 1 week after hepatectomy, which may be associated with complete removal of the tumor (Fig. 4F). Therefore, the combination scheme for CTC sorting presented in this study can predict HCC progression and can be further applied to clinical research. In vivo results showed that CTC counts increased sharply with the occurrence of lung metastasis at the fifth week of tumor inoculation in situ, which was equivalent to HCC patients at a clinically advanced stage, suggesting that the combination scheme might achieve a good detection effect in advanced patients. Then, HCC patients with TNM stage IIIc-IV were recruited for the study.

### Identification of circulating tumor cells in HCC patients

**Fig. 5:**
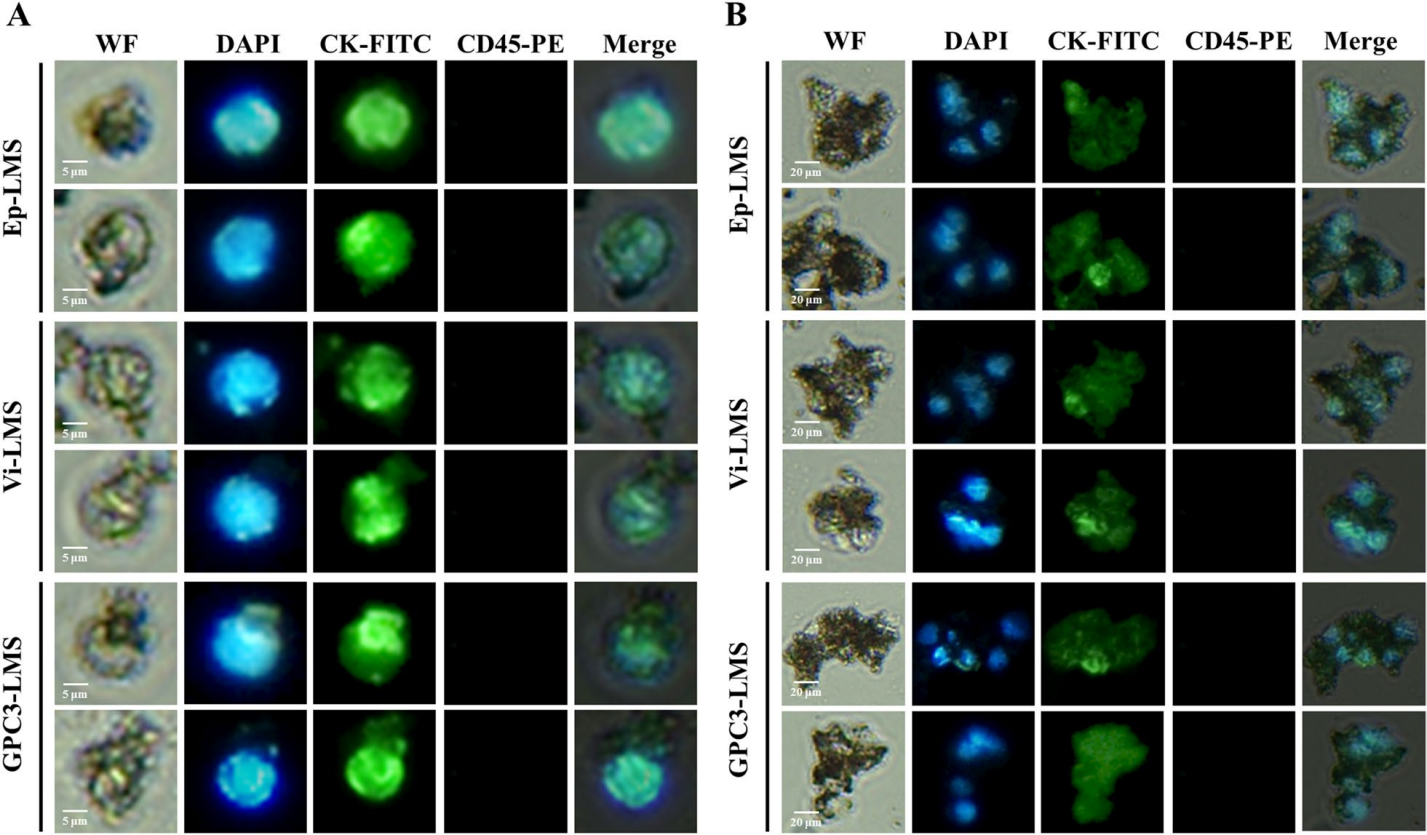
Identification of CTCs via immunofluorescence by DAPI, CK-FITC, and CD45-PE for HCC patients. **A** Identification of single CTCs. **B** Identification of CTC clusters

The separated CTCs (Fig. 5A) and CTC clusters (Fig. 5B) were smeared on a slide and observed using a fluorescence microscope. When cells were visible on the brightfield setting, a green fluorescence filter was used to identify CK-FITC-positive tumor cell membrane. A blue fluorescence filter was used to identify DAPI-positive nuclei. A red fluorescence filter was used to exclude CD45-PE-negative leukocytes. Therefore, the tumor cells with CK^+^/DAPI^+^/CD45^−^ and obvious cellular morphology were identified as CTCs.

### Clinical practice further validates the in vitro and in vivo findings of the Ep-LMS/Vi-LMS/GPC3-LMS system

**Fig. 6:**
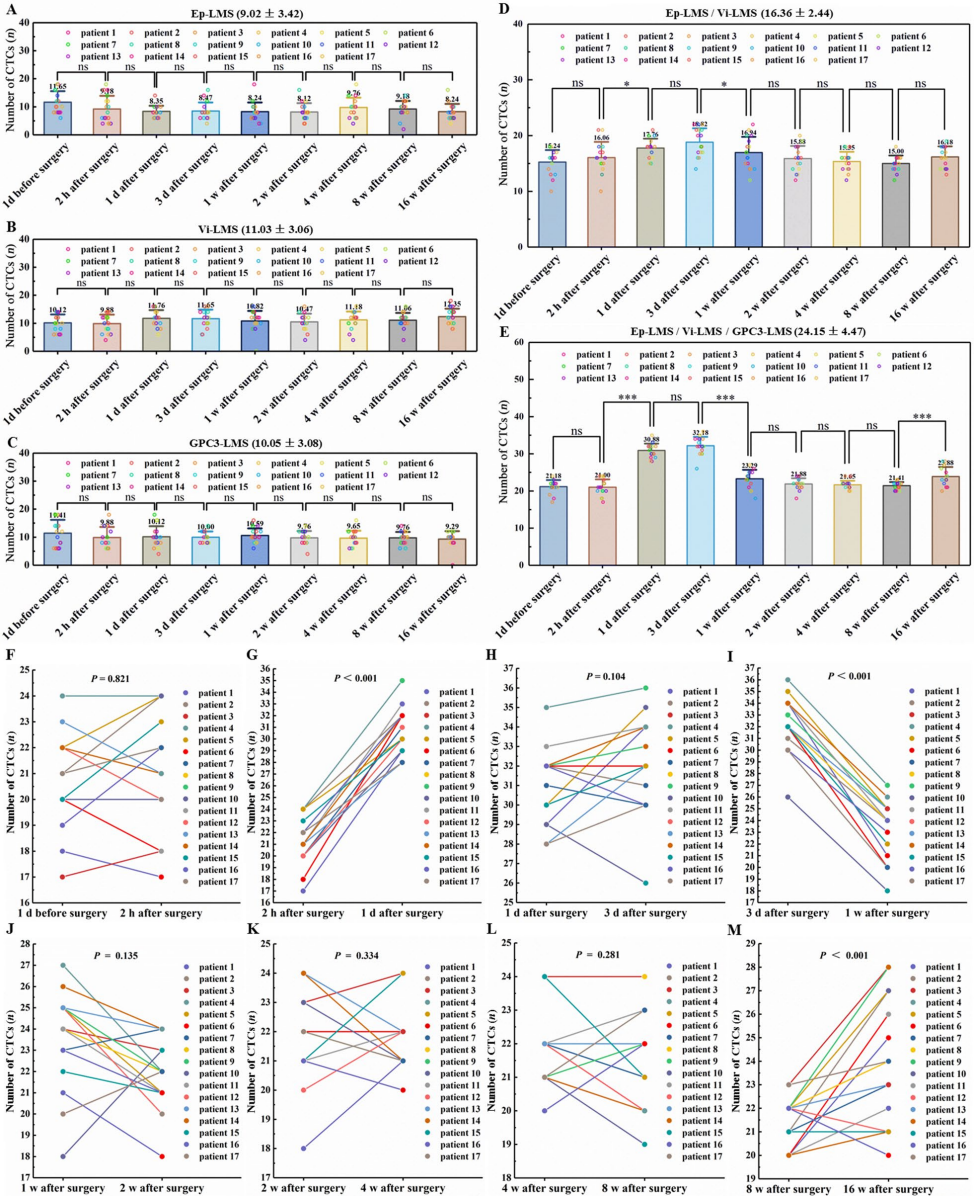
Dynamic changes in the number of CTCs. **A**–**E** Distribution of CTCs captured by Ep-LMS, Vi-LMS, GPC3-LMS, Ep-LMS/Vi-LMS, and Ep-LMS/Vi-LMS/GPC3-LMS. **F**–**M** Number of CTCs captured by Ep-LMS/Vi-LMS/GPC3-LMS at different time points. One asterisk (*) indicates a significant difference (*P* < 0.05); three asterisks (***) indicate a significant difference (*P* < 0.001); “ns” indicates no significant difference

During multidisciplinary comprehensive treatments, 3 patients withdrew from the HCC group, 13 cases had hepatectomy following conversion therapy (13/17, 76.5%), and 4 cases with TACE could not receive liver resection due to failed comprehensive therapies (4/17, 23.5%). Therefore, 17 patients with advanced HCC included in this study were followed up, and CTCs were detected at different time points, as shown in Fig. 6. The number of CTCs caught by Ep-LMS, Vi-LMS, GPC3-LMS, and Ep-LMS/Vi-LMS was not significantly different (Fig. 6), which further validated the in vivo results. The mean CTC counts were 9.02 ± 3.42/2 mL, 11.03 ± 3.06/2 mL, 10.05 ± 3.08/2 mL, and 16.36 ± 2.44/2 mL, respectively (Fig. 6A–D). The expression ratio of EpCAM, vimentin and GPC3 in CTCs was 1.00:1.22:1.11. The average number of CTCs captured using the Ep-LMS/Vi-LMS/GPC3-LMS sequential capture strategy was 24.15 ± 4.47/2 mL, while the preoperative mean was 21.18/2 mL. Two neighboring groups had very different numbers of CTCs (the presence of three asterisks means *P* < 0.001, Fig. 6E). The CTC counts increased 1 day after surgery (*P* < 0.001, Fig. 6G), reached the highest level 3 days after surgery (Fig. 6H), decreased significantly 1 week later (*P* < 0.001, Fig. 6I), and increased again 16 weeks after hepatectomy (*P* < 0.001, Fig. 6M). Other comparisons revealed no significant differences (all *P* > 0.05; Fig. 6F, H and J–L).

No CTCs were detected in the controls. Clinical correlation analyses revealed that the number of CTCs before surgery was significantly correlated with tumor emboli (*P* = 0.002) and tumor metastasis (*P* = 0.004, Table 1). We also observed that the CTC count within three days after surgery was positively correlated with the numbers of leukocytes, monocytes, and neutrophils and negatively correlated with the AFP level (Additional file 9: Table S1). After sixteen weeks of postoperative follow-up, the number of CTCs increased suddenly in six patients; this was closely related to intrahepatic tumor recurrence (one case), drug withdrawal due to drug eruption and diarrhea (four cases), preoperative spontaneous tumor rupture (intrahepatic recurrence was confirmed 60 weeks after liver resection) and postoperative excessive fatigue (one case). Considering the patient’s physical condition, a sudden increase in the CTC count may suggest a risk of tumor progression. These findings indicate that the combination scheme for dynamic CTC detection could be applied to sensitively evaluate the efficacy of treatment and monitor HCC progression, implying the importance of timely, effective and positive interventions after resection.

**Table 1:** Correlation of clinicopathological parameters with the number of preoperative CTCs in HCC patients

Clinicopathological parameters	Cases (*n*)	Number of preoperative CTCs		*P*
		Mean	SD	
Age (years)	17			0.285
≤ 65	7	20.67	2.05	
> 65	10	21.60	1.20	
Sex	17			0.429
Male	13	21.03	1.81	
Female	4	21.83	0.55	
Cirrhosis	17			0.566
Yes	13	21.08	1.84	
No	4	21.67	0.75	
Tumor emboli	17			0.002
Yes	9	22.30	0.84	
No	8	20.00	1.53	
Bile duct invasion	17			0.826
Yes	5	21.07	1.55	
No	12	21.28	1.71	
Bile duct hyperplasia	17			0.358
Yes	4	20.50	1.52	
No	13	21.44	1.65	
Immunotherapy	17			0.647
Yes	14	21.26	1.84	
No	3	21.11	1.26	
Metastasis	17			0.004
Yes	10	21.90	1.04	
No	7	19.57	1.59	

*HCC* hepatocellular carcinoma; *CTCs* circulating tumor cells; *Mean* the mean value; *SD* standard deviation

### CTCs captured using the combination scheme share mutations with cells in tumor tissues and sensitively predict immunotherapy benefit

**Fig. 7:**
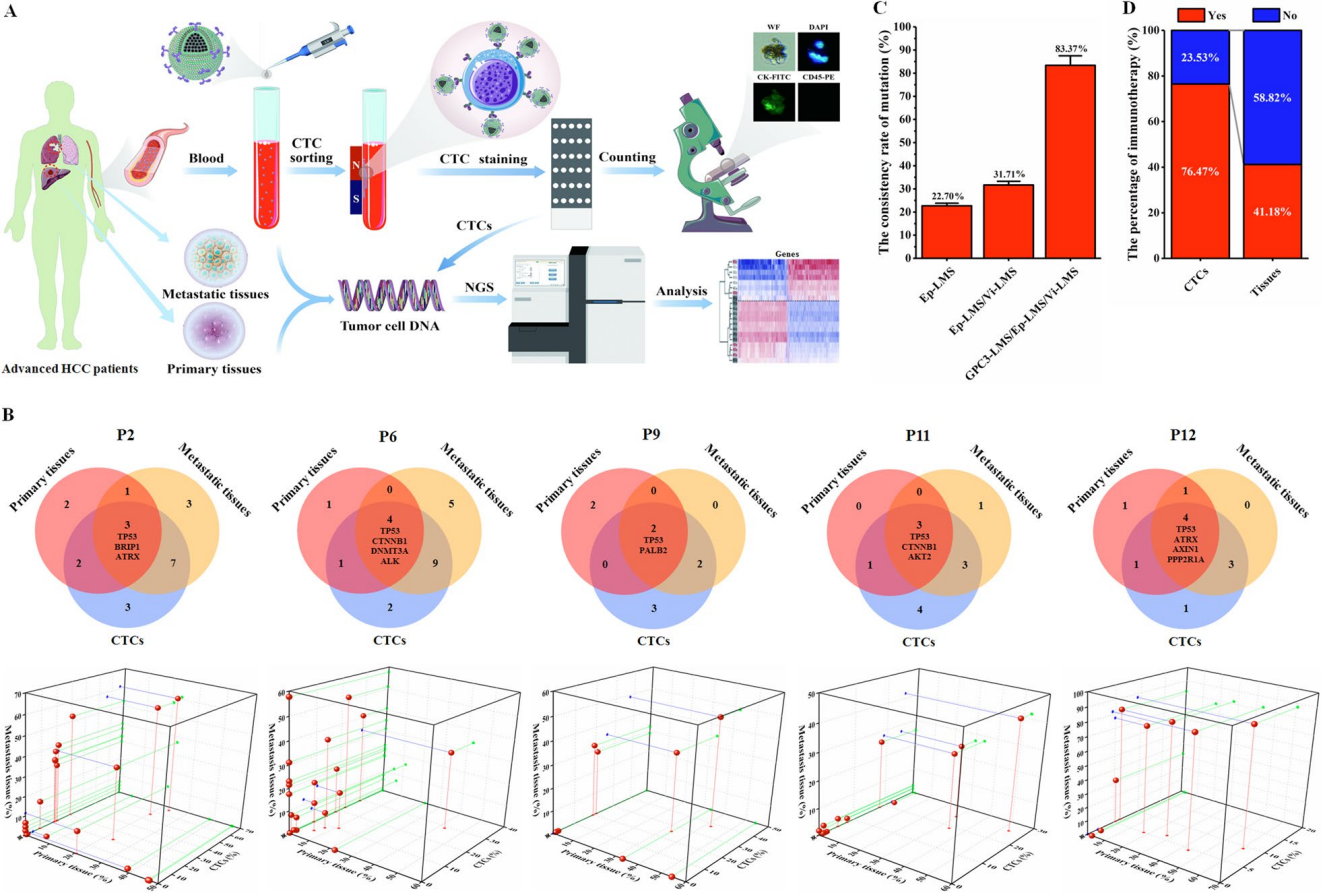
Detection of gene mutation and its clinical significance. **A** Flow chart of gene detection for HCC patients. **B** Gene mutations in primary tissues, metastatic tissues and CTCs of five patients (P2, P6, P9, P11, P12). **C** CTCs shared gene mutations consistent with those of metastatic tissues. **D** Percentage of immunotherapy between groups

In this study, multiple subsets of CTCs were captured from HCC patients by the combination scheme of Ep-LMS/Vi-LMS/GPC3-LMS. To assess the suitability of CTCs in analyzing the tumor mutational landscape, we conducted NGS on matched CTC samples and tumor tissues from 17 HCC patients. Figure 7A shows a flow chart, and the 11 genes marked in red have alterations with therapeutically targeted medications (Additional file 7: Fig. S7A–C). Mutations in the *TP53*, *ALK*, and *ATRX* genes, the genes most frequently mutated in both HCC primary tissues and CTCs, were observed in 59%, 24%, and 18% of patients, respectively, and *TP53*, *ARID1A*, *CDKN2A*, *CTNNB1*, *BRIP1*, *EGFR*, *FGFR2*, *RB1*, and *TSC1* gene mutations with clinical targeted drugs were detected in primary tissues and CTCs from the same patient (Additional file 1: Fig. S7D, *n* = 17). As seen from the above results, both primary tissues and CTCs could detect mutations in targetable genes with clinical significance. The number of gene mutations in CTCs was lower than that in primary tissues, which may be due to the greater number of tumor cells in tissue samples. However, more high-frequency gene mutations were found in CTCs.

In fact, CTCs can disseminate from both the primary tumor and secondary tumor and share some mutations with tumor tissues. To better illustrate the clinical value of more subpopulations of CTCs captured by the scheme, differential analysis of mutations was performed among the matched primary tumor tissues, CTCs and metastatic tissues from 5 HCC patients (P2, P6, P9, P11, and P12). The results showed that *TP53*, *CTNNB1* and *ATRX* were the most commonly mutated genes (Fig. 7B). Furthermore, when we compared gene mutations in CTCs captured by the Ep-LMS, Ep-LMS/Vi-LMS and Ep-LMS/Vi-LMS/GPC3-LMS techniques, we found that the gene mutations of CTCs captured by the combination scheme (Ep-LMS/Vi-LMS/GPC3-LMS sequential capture scheme) were highly consistent with those of metastatic tissues (83.37%, Fig. 7C). The above results indicate that CTCs share mutations with primary and metastatic lesions, which may help guide HCC therapy. Regarding tumor immunotherapy, the proportion of patients predicted to benefit from immunotherapy based on the CTC detection method was greater than that based on the tissue detection method (13/17, 76.47% vs. 7/17, 41.18%, *P* = 0.037, Fig. 7D; Additional file 8: Fig. S8); 14 HCC patients accepted immunotherapy, which shows that CTCs might be employed as a suitable alternative to tumor tissues. In addition, the results of follow-up showed that the immunotherapy group had a lower percentage of tumor progression than the nonimmunotherapy group (4/14, 28.57% vs. 2/3, 66.67%). The above results validated that the combination scheme (Ep-LMS/Vi-LMS/GPC3-LMS) is convenient and feasible in clinical practice, and CTCs captured by this method have many advantages, which facilitates the clinical application of CTC liquid biopsy.

## Discussion

HCC is a common and highly malignant tumor. Without timely diagnosis and treatment, the survival time of primary liver cancer patients may be only 3–6 months. Surgical resection is one of the most effective methods for HCC. However, the liver has a rich blood supply and is prone to metastasis after hepatectomy due to hematogenous metastasis. Moreover, when the portal vein is invaded by tumor cells, tumor thrombi will form, resulting in focal metastasis. Additionally, the entry of tumor cells into systemic circulation through the blood will lead to extrahepatic metastasis. To limit such metastases, effective prevention, treatment, and monitoring of tumor cell metastasis are vital.

In recent years, CTCs have attracted extensive attention as potential “seeds” of tumor metastases. CTCs play an important role during tumor metastasis [23]. The bottleneck of clinical CTC application lies in enrichment technology. At present, there are various CTC separation and enrichment strategies, but each has its own advantages and disadvantages, as shown in Additional file 10: Table S2. The US FDA approved CTC count as a prognostic indicator (CellSearch method) for patients with advanced metastatic breast cancer, prostate cancer, and colorectal cancer (with CTC values greater than 5, 5, and 3/7.5 mL of whole blood, respectively, indicating a poor prognosis). The basic principle of CTC acquisition is based on EpCAM antibody capturing epithelial phenotype CTCs. This method has great limitations in clinical application in HCC CTC detection, and its sensitivity is relatively low (20–40%) [24, 25]. We previously constructed a CTC sorting system, and its capture efficiency was 4.4 ± 1.2 times higher than that of the CellSearch system after coupling with EpCAM [26]. However, it is still not enough to use EpCAM or vimentin alone or both in CTC isolation. For HCC, the separation scheme of EpCAM and vimentin combined with GPC3 can be used for more complete separation of CTCs, which improves the detection sensitivity [22, 27]. For the first time, we designed and constructed a CTC sorting system with clinical feasibility based on Ep-LMS/Vi-LMS/GPC3-LMS according to three different molecular types (the combination scheme), including the EMT phenotype and GPC3 phenotype specifically expressed in HCC. After characterization, in-depth research and preliminary verification were carried out in cell models, animal models, and clinical samples. The results showed that the combination scheme has the advantages of low toxicity, strong specificity (96.94%), high sensitivity (98.12%) and a high detection rate (98.64%), which can specifically, accurately and efficiently enrich CTCs in blood, with an average number of CTCs of 24.15 ± 4.47/2 mL.

Hepatectomy is still the most effective treatment for HCC, and CTC counts can be used as an effective method in evaluating postoperative efficacy and monitoring tumor progression [28, 29]. Preoperative CTC analyses can better guide the resection of HCC. Qi et al. [30] compared the prognoses of patients undergoing anatomical resection and nonanatomical resection according to the number of CTCs and EMT phenotype. The results showed that anatomical resection could improve the survival rate of HCC patients with a small number of CTCs, epithelial/mesenchymal phenotype(s), and no mesenchymal CTCs. Moreover, CTC detection can also provide a reference for the evaluation of postoperative efficacy in patients with HCC. Several clinical studies [31, 32] have shown that the significant decrease in the CTC count after surgical resection can reflect high efficacy, while the increase in the CTC count after surgery is related to the deterioration of prognosis. Furthermore, the dynamic changes in CTC counts can reflect the efficacy in patients. Rau et al. [33] confirmed the clinical utility of continuous CTC monitoring in patients with locally advanced or metastatic HCC receiving systemic/targeted therapy. In most cases, the changes in CTC counts are related to efficacy in patients and have a guiding role for patients with negative AFP. Yun-Fan Sun et al. [34] studied the spatial heterogeneity of CTCs using animal models, mapped the transmission route and characteristics of CTCs in patients with localized tumors, and proved that CTCs had obvious heterogeneity in number, size, and clustering, confirming that the activation of EMT programs in CTCs mainly occurs during migration. In this study, we found that a high number of preoperative CTCs was significantly correlated with tumor emboli and metastases. An increase in CTC count within 3 days after surgery was positively correlated with the number of leukocytes, monocytes, and neutrophils, which may be associated with operation, anesthesia trauma, and patient’s damaged immune system function. Then, the number of CTCs decreased significantly, which may be attributable to the gradual rehabilitation of postoperative physiological function. The subsequent decreased AFP level confirms the efficacy of hepatectomy. During follow-up, we found that CTC counts suddenly increased in 6 patients 16 weeks after resection due to recurrence, drug withdrawal, and preoperative spontaneous tumor rupture (diagnosed intrahepatic recurrence by reresection 60 weeks after hepatectomy). Whether the increase in the number of CTCs is a prelude to recurrence and metastasis still needs to be verified by further follow-up and multicenter clinical studies. However, these findings prove for the first time that the combination scheme can monitor the dynamic changes in CTC counts in advanced HCC patients more sensitively than single-method schemes, providing a sensitive method for effectively monitoring tumor progression.

An expanding body of evidence indicates the efficacy of immunotherapy in both second-line and first-line settings for advanced HCC. Therefore, it is necessary to detect corresponding mutated genes and then select the most effective therapeutic schedule. Compared with traditional detection methods, NGS has the advantage of offering a single-time high-throughput detection of the status and changes of multiple genes in vivo, enriching the rigid requirements for clinical diagnosis, treatment, and continuous monitoring of HCC prognosis [35, 36]. Biopsy is the gold standard diagnostic method for solid tumors. However, clinical complications and technical difficulties associated with biopsy are obstacles to the acquisition of tissue samples in subsets of HCC patients. CTCs represent viable cells with metastatic potential in the circulatory system. The clinical utility of the genomic analysis of CTCs has not been fully demonstrated thus far, partially as a result of the low yield of DNA from these cells, which presents challenges for library preparation and bioinformatics analysis in NGS. Our results showed that CTCs shared partial mutations with primary tumors, which can be an appropriate alternative when tissue samples are difficult to obtain in clinical practice. Moreover, CTC gene detection can be used as an effective complement to tissue detection, providing a valuable data reference for the clinical diagnosis and treatment of HCC.

It has been shown that tumor mutation burden (TMB) is related to the effectiveness of immunotherapy; the higher the TMB is, the greater the tumor inhibition effect and clinical benefits achieved from immunotherapy; conversely, the lower the TMB is, the worse the clinical benefits acquired from immunotherapy [37]. In this study, we found that CTC sequencing is more sensitive than tissue sequencing in terms of identifying patients who could benefit from immunotherapy. Genetic testing and analysis of both tumor tissues and CTC samples from the same patient could increase the accuracy of gene detection could to guide clinical immunotherapy more accurately, thereby improving patient prognosis. Furthermore, high similarity of gene mutations was also found between metastatic tissues and CTC samples, indicating that CTC gene detection could provide a mutational atlas for metastatic HCC. Therefore, the CTCs captured by the combination scheme have potential value in predicting metastasis and suggesting the benefits from immunotherapy, which promotes the application of CTC liquid biopsy with clinical significance. Moreover, the rapid detection and quantification of CTCs is a key need. It is necessary to develop supporting software and algorithms to support automatic focusing of the microscope, coordinate positioning of target cells, intelligent recognition and interpretation. These works emphasize difficulties in the future development of CTC detection technology, which must rely on the deep integration of biology, optics, mechanics, software development and artificial intelligence to achieve breakthroughs.

## Conclusions

In this study, EpCAM/vimentin/GPC3 antibody-modified LMS were used to capture tumor cells with epithelial phenotype, mesenchymal phenotype and GPC3 phenotype. For the first time, a therapeutically practical CTC sorting method based on sequential enrichment of Ep-LMS/Vi-LMS/GPC3-LMS (the combination scheme) was established, and this method captures several subpopulations of CTCs effectively and sensitively for dynamically monitoring HCC progression. In addition, this study confirms that the gene mutations in matched primary tissues, CTCs and metastatic tissues are complementary, CTCs and metastatic tissues have a higher rate of shared mutations, and gene detection in multiple subsets of CTCs can provide a more comprehensive mutation spectrum for HCC, which provides a solid foundation for clinical immunotherapy and, thus, facilitate liquid biopsy-based applications.

## Methods

### Clinical specimens

A total of 44 participants were considered for inclusion in this study from May 2021 to September 2022, including 24 healthy volunteers without any hepatitis (blank control) and 20 advanced HCC patients evaluated by clinical history, AFP and imaging findings. The inclusion criteria for HCC patients in this study were (a) availability of CTCs, frozen biopsy and/or resected primary and metastatic HCC tissues; (b) pathologically proven HCC based on WHO criteria; (c) gene mutation detection for CTCs and HCC tissues; and (d) availability of follow-up data. Advanced HCC patients received conversion therapies, including immunotherapy, targeted therapy (sorafenib/lenvatinib), transcatheter arterial chemoembolization (TACE), traditional Chinese medicine (Huaier granule) [38], and liver protection therapy. The therapeutic schedule was permitted to be adjusted according to the actual condition of individuals. Tumor samples were collected by resection or biopsy, and 10 mL blood samples were obtained each time for CTC counts at different time points: 1 day before surgery and 2 h, 1 d, 3 d, 1 w, 2 w, 4 w, 8 w, and 16 w after surgery. The matched specimens (*n* = 39), including primary lesions of HCC (*n* = 17), preoperative CTC samples (*n* = 17) and metastatic tissues (*n* = 5), were used for 610 gene mutation detection by NGS. HCC patients were followed up every month until September 30, 2022, by monitoring serum AFP levels, abdominal ultrasonography, chest X-ray or computed tomography depending on the patient’s condition. General data, metastatic characteristics, pathologic characteristics and survival were compared among the groups. This study was approved by the ethics committee of our hospital (No. 2022-020; China Clinical Trial Registration Center-Registration No. ChiCTR2200055847), and informed consent was obtained from participants in accordance with respective committee regulations.

### Consumables and instruments

Fe_3_O_4_ solution, carboxymethyl chitosan hexadecyl quaternary ammonium salt (HQCMC), CK8/18/19 (CK)-FITC (Fluorescein Isothiocyanate), CD45-PE, DAPI, Ep-LMS, and Vi-LMS were purchased from Huzhou Lieyuan Medical Laboratory Co., Ltd. A Prussian blue staining kit was purchased from Solarbio. DMEM, fetal bovine serum and trypsin were purchased from Gibco, and GPC3 antibody was purchased from Abcam. 1,2-Dioleoyl phosphatidylcholine (DOPC), dimethyl octadecyl epoxy propyl ammonium chloride (GHDC), cholesterol, dichloromethane, N-hydroxysuccinimide (NHS), 1-ethyl-3-(3-dimethylaminopropyl)-carbodiimide (EDC) and other commonly used reagents were all purchased from Sinopharm Group. The TIANamp Genomic DNA Kit was purchased from Tiangen Biotech (Beijing).

### Cell lines and culture

At the authors’ institution, a stepwise metastatic human HCC model system was established, which included a metastatic HCC model in nude mice (LCI-D20) [39], an HCC cell line with high metastatic potential that originated from LCI-D20 tumor (MHCC97) [40], a highly metastatic subclone (MHCC97H with a lung metastasis rate up to 100% using orthotopic inoculation) and a subclone with lower metastatic potential (MHCC97L with a pulmonary metastasis rate up to 40% using orthotopic inoculation) established through in vivo selection of MHCC97 cells [41]. The HCC cell lines Hep3B and Huh7 with low metastatic potential and human umbilical vein endothelial cells (HUVECs) were purchased from the Shanghai Cell Bank of the Chinese Academy of Sciences. The cell lines were cultured following procedures stated in our previous reports [42]. HUVECs were grown in RPMI-1640 medium (Gibco-BRL, Gaithersburg, MD, USA) supplemented with 10% fetal bovine serum (HyClone, Logan, UT, USA). The remaining cell lines were maintained in antibiotic-free Dulbecco’s modified Eagle’s medium (DMEM, Gibco-BRL, Gaithersburg, MD). All cell lines were cultured without antibiotics in a humidified incubator containing 5% CO_2_/95% air at 37 °C.

### Preparation of GPC3 magnetic spheres

Cholesterol (6 mg), DOPC (500 µL, 10 mg/mL) and emulsifier HQCMC (500 µL, 10 mg/mL) were codissolved in dichloromethane as the solvent. Then, 1 mL 20 mg/mL Fe_3_O_4_ solution and 20 mL 0.1 mol/L phosphate buffer saline (PBS) (pH 7.4) were added, followed by ultrasonic oscillation under a power of 100 W at 25 °C for 6 min and rotary evaporation for 30 min to remove dichloromethane and obtain LMS solution (1.8 mg/mL). Subsequently, 0.1 mg surfactant GHDC was dissolved in 1 mL isopropyl alcohol, and 60 µg GPC3 was dissolved in 1 mL GHDC solution (GPC3-GHDC), which was added to the coupling agents NHS (0.1 mg) and EDC (0.1 mg). After incubation overnight at 4 °C, 1 mL GPC3-GHDC was added into 1 mL LMS solution, which was subjected to vortex oscillation for 5 min and stored at 4 °C. Finally, the sample was taken out of the vortex every 1 h for 5 min for 24 h to obtain GPC3-LMS (2.16 mg/mL).

### Characterization

The sample (10 μL) was diluted in 1 mL of distilled water, and the particle size and potential of the magnetic spheres were measured by using a BI-90Plus laser particle sizer/Zeta potentiometer (Brookhaven), 50 μL of which was coated on mica plates. The morphology of LMS was observed under an AFM. Then, 50 μL of diluent was dropped on a copper mesh, and the morphology of LMS was observed under TEM after drying. After freeze-drying of a 1 mL sample, the infrared spectrum obtained by the KBr tablet method was measured on a Bio-Rad FTS 6000 spectrometer using FT-IR spectroscopy. Afterward, 10 μL samples were taken for magnetic separation and diluted in 1 mL distilled water, and the LMSwere scanned by ultraviolet absorption spectroscopy with an ultraviolet spectrophotometer. After a 1 mL sample was freeze-dried into powder, the magnetization curve was determined using a vibrating sample magnetometer (VSM) at room temperature.

### Cytotoxicity assay

The cells were prepared into a single-cell suspension with trypsin and cultured in 96-well plates at 8000 cells per well. When the cells grew to 80% confluence, the medium was replaced with 100 μL of complete DMEM containing different amounts of Ep-LMS, Vi-LMS and GPC3-LMS. In the blank control group, 100 μL of complete DMEM was added for further culture. After adding 20 μL MTT reagent (5 mg/mL), incubation was performed in a CO_2_ incubator for 3 h. Finally, 150 μL dimethyl sulfoxide (DMSO) was added to dissolve the prepared blue and purple crystalline formazans. The experimental results were read in a microplate reader and counted (wavelength, 490 nm), and 3 parallel tests were carried out in each group.

### Distribution of magnetic spheres

Single-cell suspensions of Hep3B, Huh7, MHCC97L and MHCC97H cells were prepared. After counting, 100 cells were added to 7.5 mL PBS solution to simulate CTC suspension by separation with LMS, Ep-LMS, Vi-LMS and GPC3-LMS. The isolated cells were then stained with Prussian blue dye and observed under a fluorescence microscope after fixation on porous slides. Additionally, the captured cells were smeared on the sample mirror, sprayed with gold after drying, and observed under a SEM.

### Cell capture efficiency

Using the Ep-LMS/Vi-LMS/GPC3-LMS capture scheme, HUVECs, Hep3B, Huh7, MHCC97L and MHCC97H cells were adjusted to different cell gradients of 10, 50, 100, 500, 1000, 5000 and 10,000 cells, respectively. The sensitivity and specificity of this scheme were determined in the PBS system and the simulated blood system, respectively. Finally, magnetic spheres with antibodies at 0, 20, 40, 60, 80 and 100 μg/mL were used to capture Huh7 cells. The cell capture efficiency of magnetic spheres was investigated in the above systems.

### Cell capture time

Huh7 cells (1 × 10^4^) were incubated in a culture dish, which was supplemented with 1 mL cell culture medium and then cultured in a constant-temperature 5% CO_2_ incubator at 37 °C for 24 h. After replacing the culture medium, 20 μL Ep-LMS-FITC or Vi-LMS-FITC, GPC3-LMS-FITC, 100 μL DAPI and 100 μL Dil were added. Finally, the culture dish was fixed on a fluorescence microscope and photographed at 0, 5, 10, 15 and 20 min.

### Mouse grouping and treatments

Male athymic BALB/c nu/nu mice of 18–20 g at 5 weeks of age were obtained from the Shanghai Institute of Materia Medica, Chinese Academy of Science. All mice were handled according to the recommendations of the National Institutes of Health Guidelines for Care and Use of Laboratory Animals. Human HCC tumor models produced by MHCC97H were established in nude mice by orthotopic inoculation, as described in our previous publications [42–45]. Blood samples were obtained for further examination, and lungs and other organs suspected of tumor involvement were sampled for HE staining. Paraffin blocks of 10% buffered formalin-fixed samples of tumor and various organ tissues were prepared, serial sections were cut at 5 μm, and lung metastatic nodules were verified with HE staining.

In the subcutaneous implantation tumor model group (*n* = 15), 3 nude mice were randomly selected at the end of weeks 0, 2, 3, 4 and 5 for weighing and then sacrificed for sample collection and CTC capture. Tumor volume was calculated by the formula *V* = *a* × *b*^2^/2, where *a* is the long tumor axis and *b* is the short tumor axis. Then, 72 mice were randomized into 4 groups (54 mice in the resection groups received orthotopic tumor transplantation, and 18 mice in the black control group only received liver exposure without tumor transplantation). Three nude mice in each group were randomly sacrificed for sample collection and CTC isolation at the end of the 0, 1st, 2nd, 3rd, 4th and 5th weeks. Resection started on Day 14 after HCC tissue implantation. In the blank control group (BC group, *n* = 18), the mice underwent liver exposure without resection; in the RR group (*n* = 18), the mice underwent radical HCC resection with a negative surgical margin; in the PR group (*n* = 18), mice underwent partial HCC resection with preservation of 2 mm of tumor pedicles [42, 44–46]; in the SO group (*n* = 18), mice underwent exposure of the liver but no resection.

### CTC isolation and identification in HCC patients

Clinical samples were collected from patients with HCC. Blood samples (10 mL) were taken at different time points, and each sample was divided into five 2 mL aliquots. Then, 15 μL Ep-LMS, Vi-LMS, and GPC3-LMS was added to the Ep-LMS group, Vi-LMS group, and GPC3-LMS group, respectively, and incubated for 15 min. The Ep-LMS/Vi-LMS group was treated with 7.5 μL of Ep-LMS and Vi-LMS successively and incubated for 15 min. The Ep-LMS/Vi-LMS/GPC3-LMS group was treated with 5 μL of Ep-LMS, Vi-LMS and GPC3-LMS successively (the combination scheme). After incubation, the centrifuge tube was inserted into the magnetic separation rack for adsorption for 10 min. Subsequently, blood was removed, and 20 μL of CK-FITC, DAPI staining solution and CD45-PE were added for staining in the dark for 15 min. After staining, washing was performed twice using 1 mL of distilled H_2_O. Finally, 20 μL of distilled H_2_O was added into the centrifuge tube and mixed well. The mixed solution was evenly coated on the anti-off slide treated with polylysine. After the droplets were naturally dried, observation and counting were carried out under a fluorescence microscope.

### Gene detection and analysis

Total DNA from tumor tissues and CTCs was extracted by the TIANamp Genomic DNA Kit (total tissue DNA > 200 ng, total CTC-DNA > 20 ng). The samples (*n* = 610) were detected via target region capture and NGS technology [22]. Furthermore, TMB was calculated and compared among groups. The reference TMB value was 3.6 Muts/Mb, and if the detection value was greater than the reference value, immunotherapy was recommended.

### Statistical analysis

Statistical analyses in this study were performed by SPSS 21.0. The data are expressed as the mean value (mean) ± standard deviation (SD). One-way ANOVA was used for comparisons between different time points, and the SNK test was used for pairwise comparisons. The results were considered statistically significant at *P* < 0.05.

## Supplementary Information

**Additional file 1: Figure S1.** Flow chart for preparation of magnetic spheres.

**Additional file 2: Figure S2.** Effects of LMS (A), Ep-LMS (B), Vi-LMS (C) and GPC3-LMS (D) on the viability of different cell lines.

**Additional file 3: Figure S3.** Optimal rendering of DAPI, FITC and Dil staining after incubation with cells for 15 min.

**Additional file 4: Figure S4.** HE staining of various organs suspected of tumor involvement in nude mice.

**Additional file 5: Figure S5.** Immunofluorescence-based identification of animal blood CTCs. CTCs were captured by Ep-LMS, Vi-LMS and GPC3-LMS and identified by immunofluorescence staining with DAPI, CK-FITC and CD45-PE.

**Additional file 6: Figure S6.** Relationship between tumor volume and the number of tumor cells.

**Additional file 7: Figure S7.** Gene mutation analysis. Heatmaps showing the frequency and distribution of gene mutations in primary tissues (A), metastatic tissues (B) and CTCs (C) and the rate of shared mutations between primary tissues and CTCs (D).

**Additional file 8: Figure S8.** TMB detection for tissues and CTCs. The reference TMB value was 3.6 Muts/Mb. If the detection value was higher than the reference value, was recommended.

**Additional file 9: Table S1.** Preoperative and postoperative clinical parameters.

**Additional file 10: Table S2. **Comparisons among different CTC enrichment techniques

## Data Availability

All data generated or analysed during this study are included in this published article [and its Additional files].
