# Supplementary figures and images for: A clinically feasible circulating tumor cell sorting system for monitoring the progression of advanced hepatocellular carcinoma
Source: J Nanobiotechnology. 2023 Jan 21;21:25. doi: 10.1186/s12951-023-01783-9 (PMC9867854; doi:10.1186/s12951-023-01783-9)

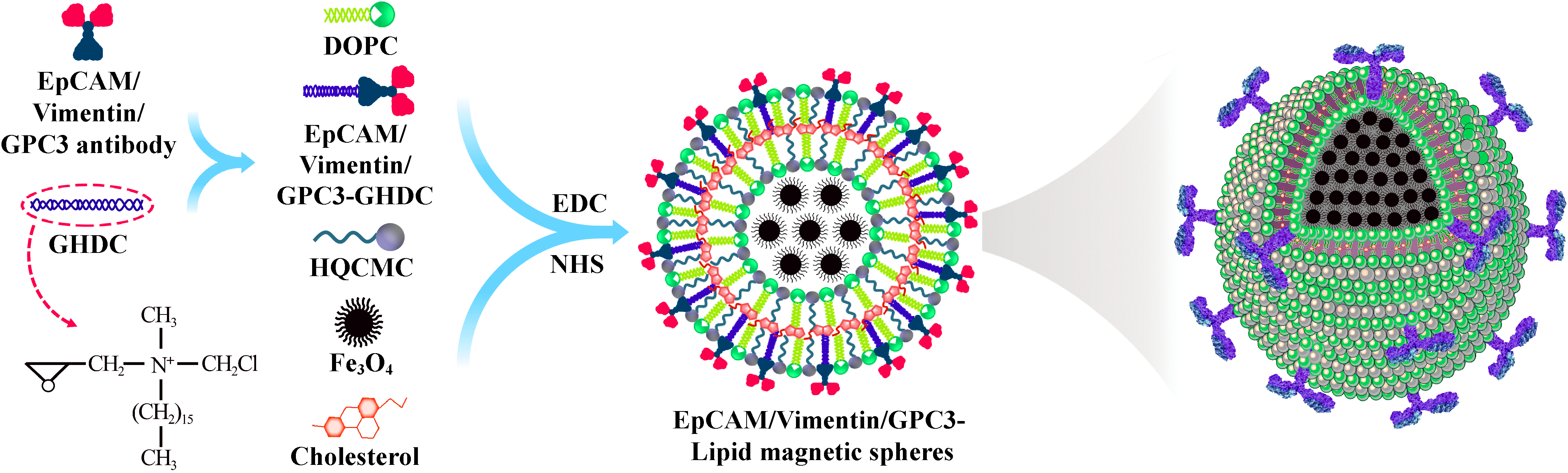

Supplement: Supplementary file 1 — Additional file 1: Figure S1. Flow chart for preparation of magnetic spheres. [file 12951_2023_1783_MOESM1_ESM.tif]

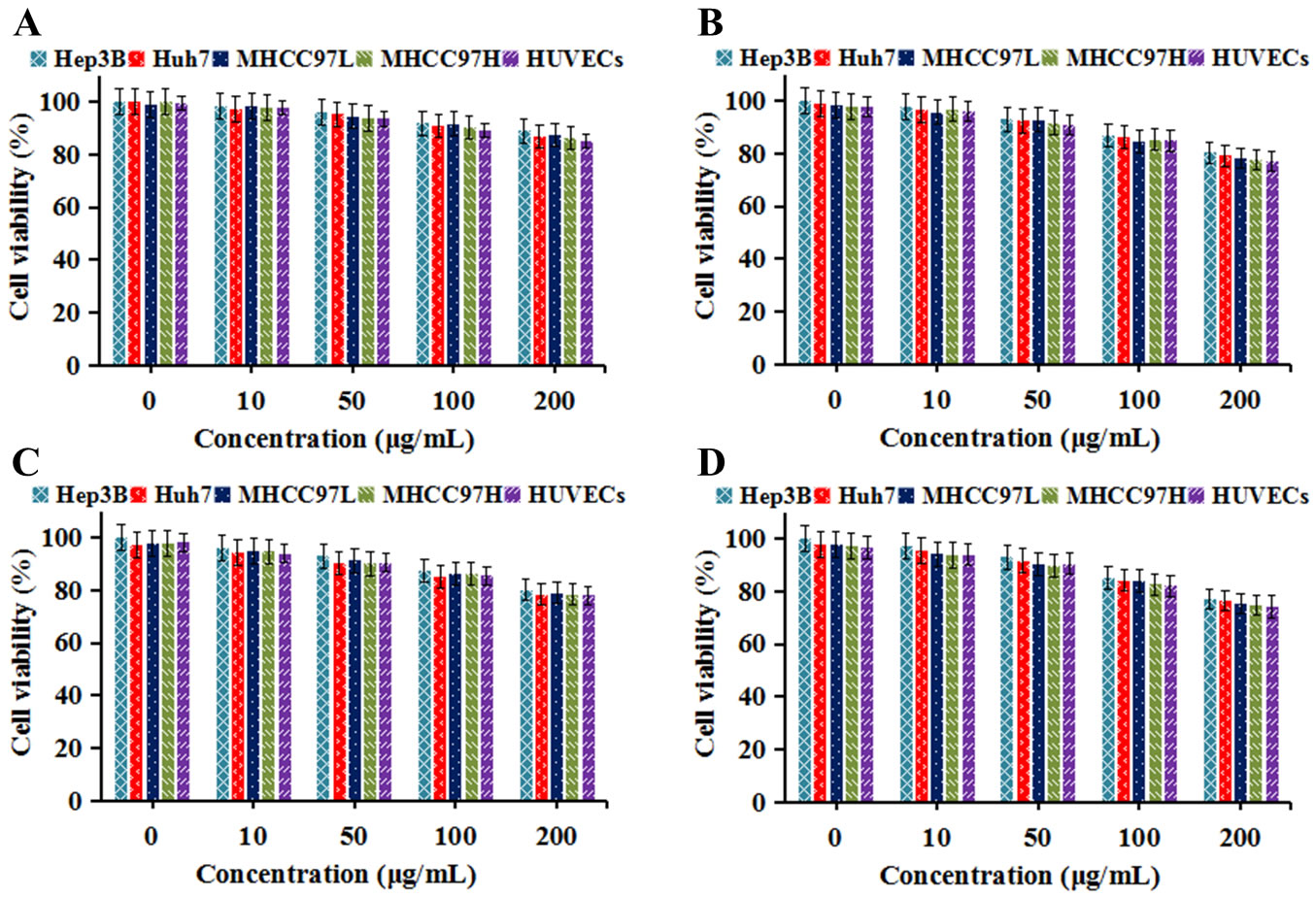

Supplement: Supplementary file 2 — Additional file 2: Figure S2. Effects of LMS (A), Ep-LMS (B), Vi-LMS (C) and GPC3-LMS (D) on the viability of different cell lines. [file 12951_2023_1783_MOESM2_ESM.tif]

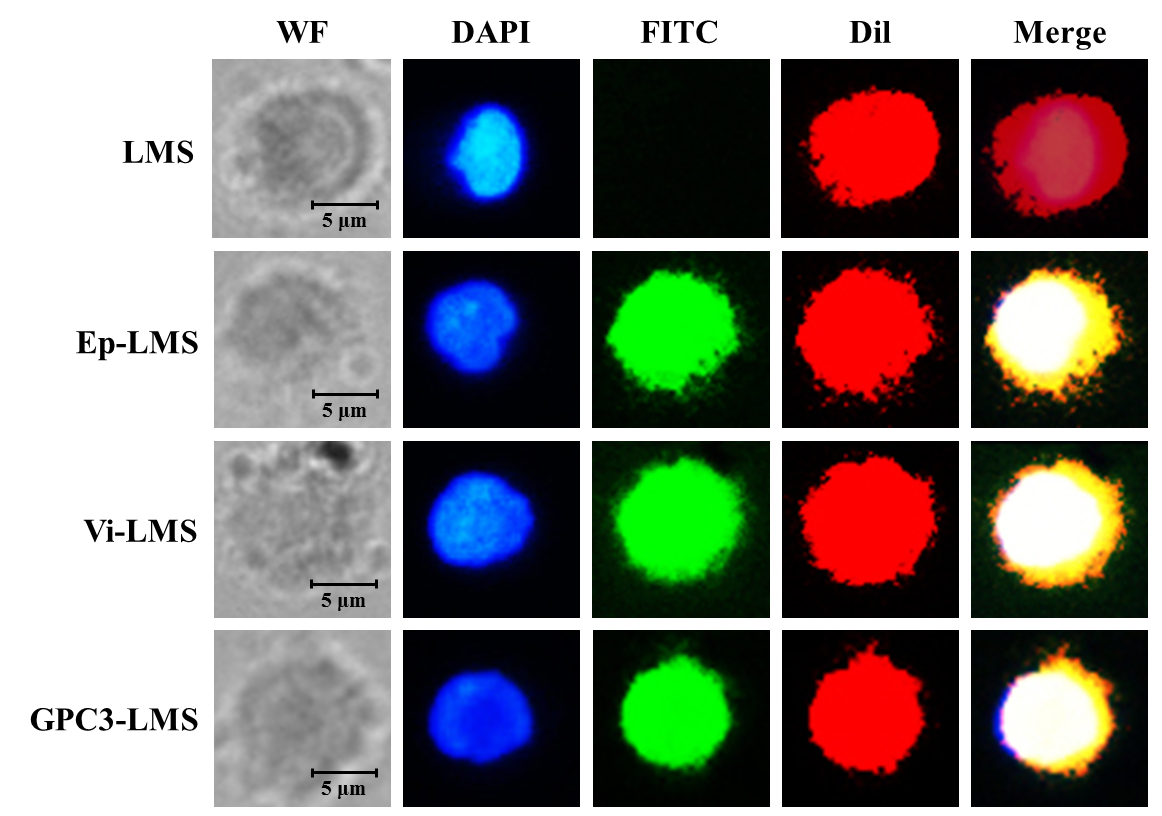

Supplement: Supplementary file 3 — Additional file 3: Figure S3. Optimal rendering of DAPI, FITC and Dil staining after incubation with cells for 15 min. [file 12951_2023_1783_MOESM3_ESM.tif]

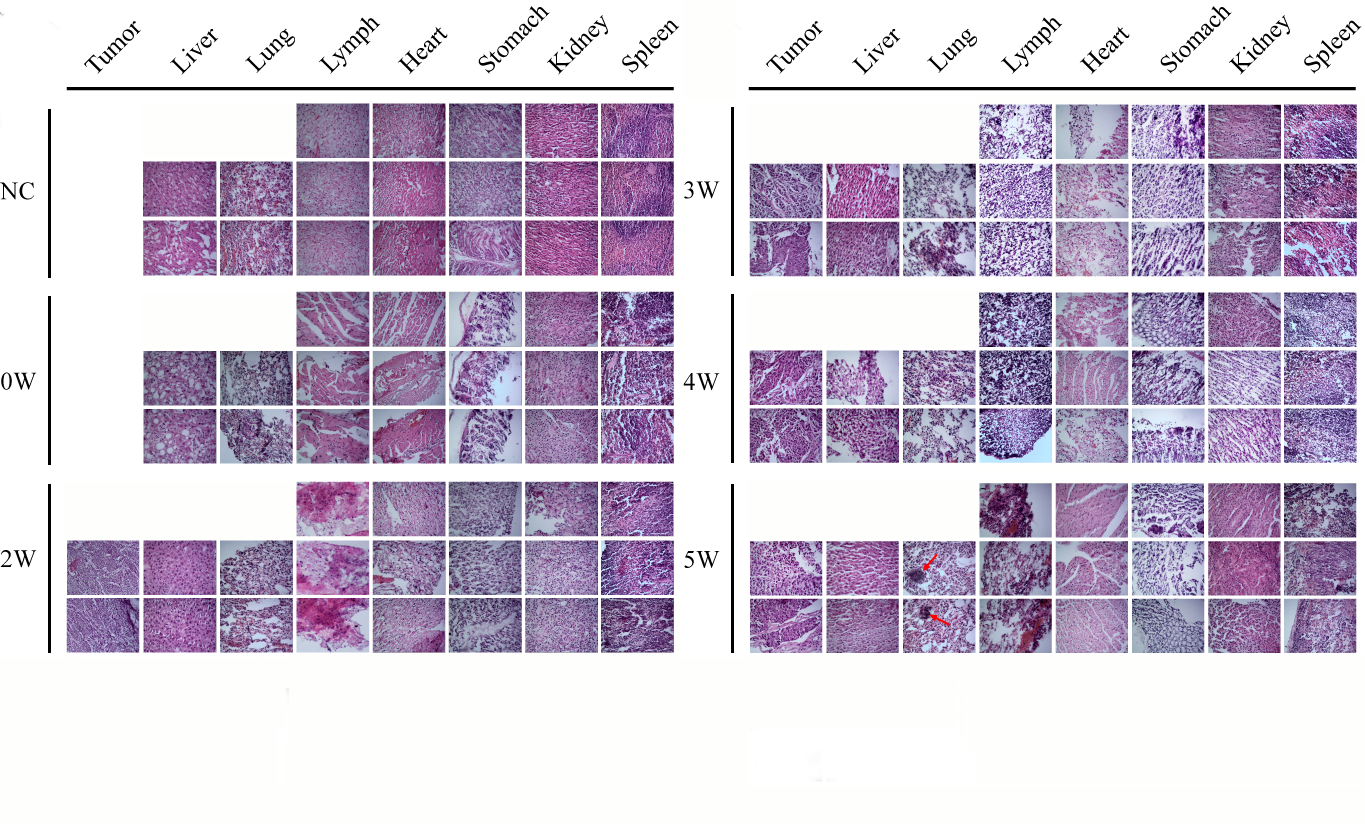

Supplement: Supplementary file 4 — Additional file 4: Figure S4. HE staining of various organs suspected of tumor involvement in nude mice. [file 12951_2023_1783_MOESM4_ESM.tif]

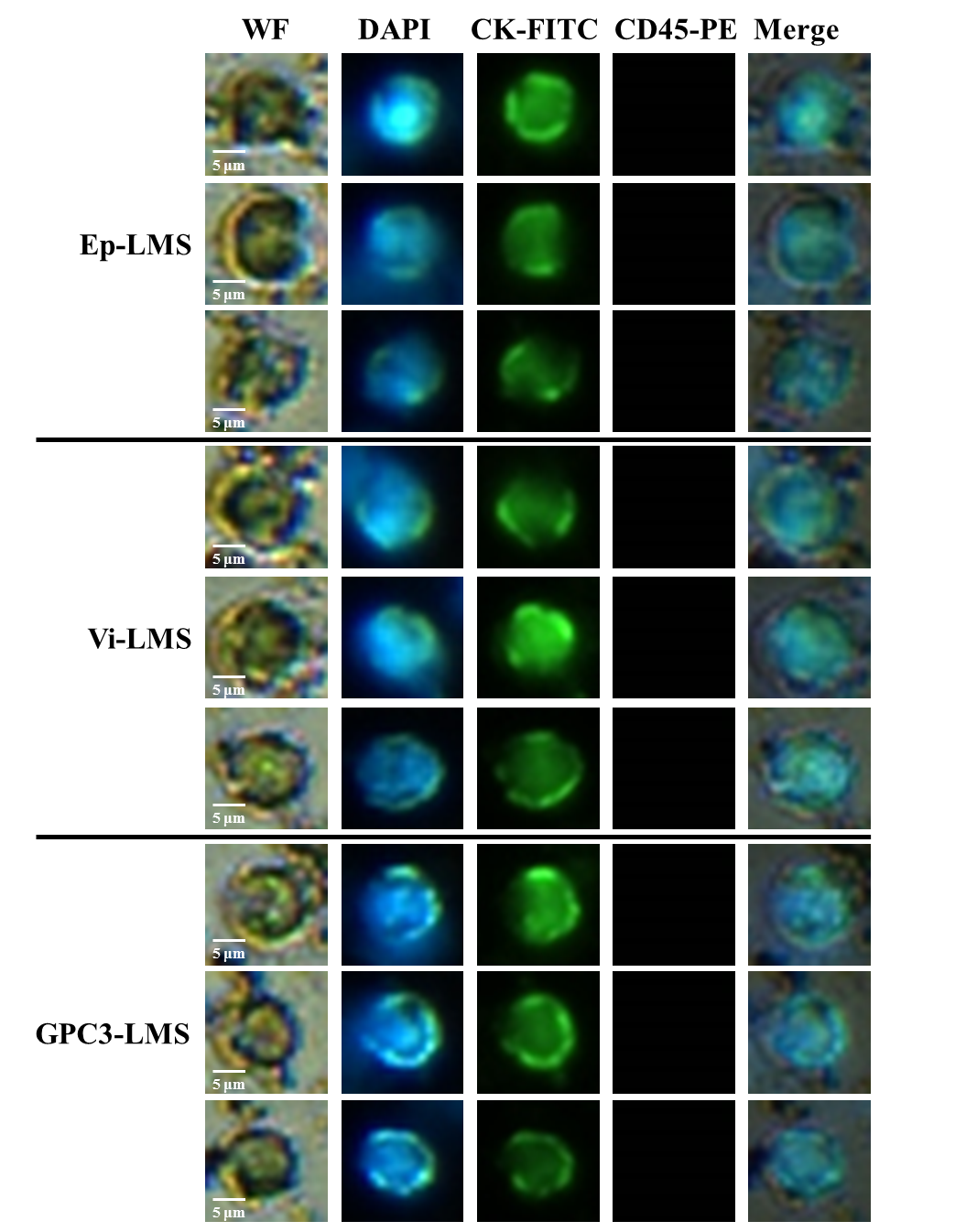

Supplement: Supplementary file 5 — Additional file 5: Figure S5. Immunofluorescence-based identification of animal blood CTCs. CTCs were captured by Ep-LMS, Vi-LMS and GPC3-LMS and identified by immunofluorescence staining with DAPI, CK-FITC and CD45-PE. [file 12951_2023_1783_MOESM5_ESM.tif]

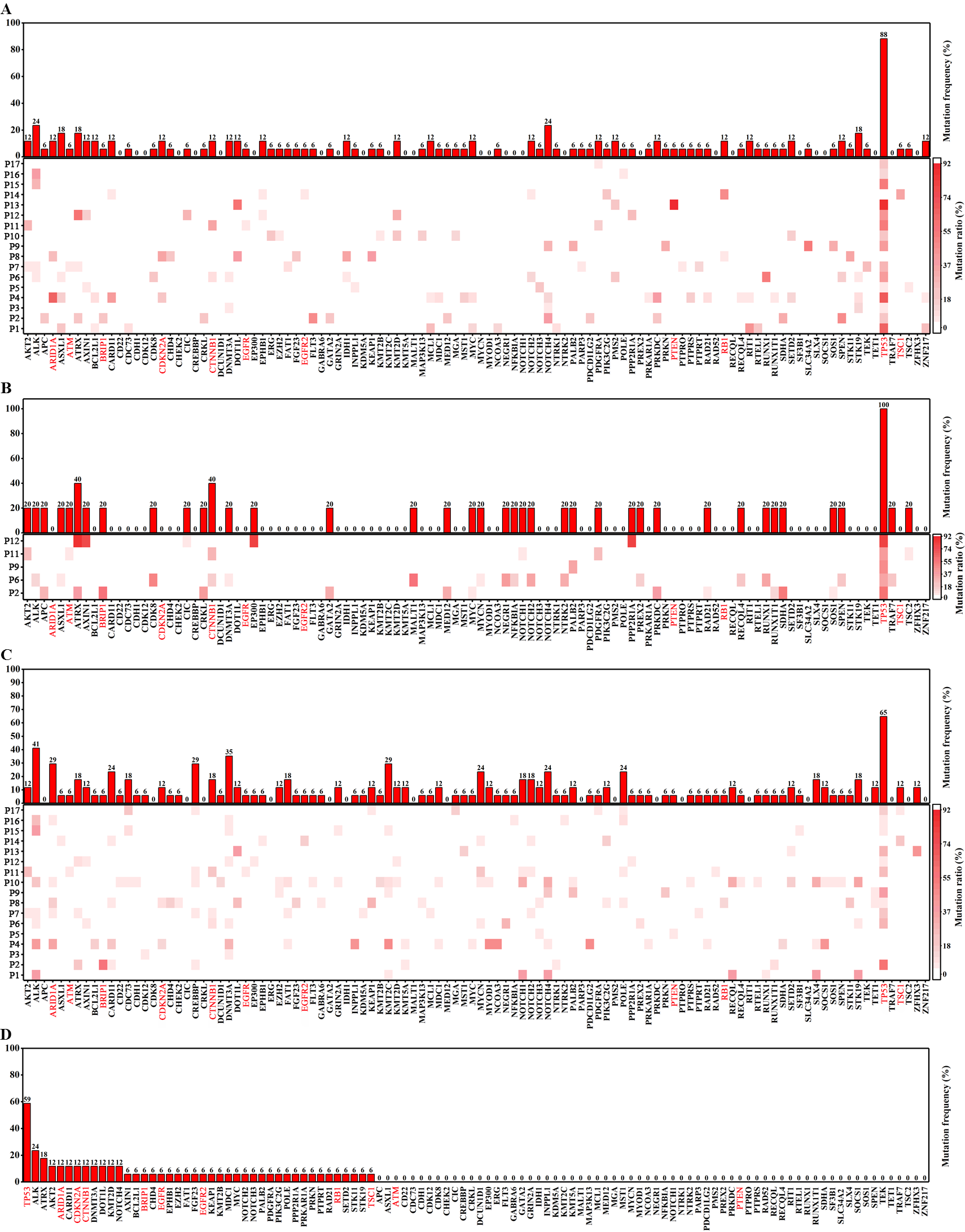

Supplement: Supplementary file 7 — Additional file 7: Figure S7. Gene mutation analysis. Heatmaps showing the frequency and distribution of gene mutations in primary tissues (A), metastatic tissues (B) and CTCs (C) and the rate of shared mutations between primary tissues and CTCs (D). [file 12951_2023_1783_MOESM7_ESM.tif]
