# Supplementary material for: A clinically feasible circulating tumor cell sorting system for monitoring the progression of advanced hepatocellular carcinoma
Source: J Nanobiotechnology. 2023 Jan 21;21:25. doi: 10.1186/s12951-023-01783-9 (PMC9867854; doi:10.1186/s12951-023-01783-9)
Supplement: Supplementary file 9 — Additional file 9: Table S1. Preoperative and postoperative clinical parameters. [file 12951_2023_1783_MOESM9_ESM.docx]

**Table S1** Preoperative and postoperative clinical parameters

| Indicators | Value (Mean ± SD) | | |
| --- | --- | --- | --- |
|  | Before surgery | 1 to 3 days  after surgery | 1 week  after surgery |
| Leukocyte (×10^9^/L) | 5.30 ± 1.08 | 17.04 ± 4.18^***^ | 7.85 ± 2.58^***^ |
| Monocyte (×10^9^/L) | 0.62 ± 0.28 | 1.52 ± 0.47^*^ | 0.86 ± 0.64^*^ |
| Neutrophil (×10^9^/L) | 3.92 ± 1.12 | 12.67 ± 2.97^**^ | 5.13 ± 2.01^**^ |
| AFP (ng/mL) | 36.15 ± 12.31 | 25.82 ± 9.62^**^ | 12.25 ± 5.36^**^ |

**NOTE:** One asterisk (*) represents there is a difference (*P* < 0.05); two asterisks (**) represent an obviously difference (*P* < 0.01); three asterisks (***) represent a significantly difference (*P* < 0.001) when compared with before surgery. Mean, the mean value; SD, standard deviation.
