# Supplementary material for: A clinically feasible circulating tumor cell sorting system for monitoring the progression of advanced hepatocellular carcinoma
Source: J Nanobiotechnology. 2023 Jan 21;21:25. doi: 10.1186/s12951-023-01783-9 (PMC9867854; doi:10.1186/s12951-023-01783-9)
Supplement: Supplementary file 10 — Additional file 10: Table S2. Comparisons among different CTC enrichment techniques [file 12951_2023_1783_MOESM10_ESM.docx]

**Table S2** Comparisons among different CTC enrichment techniques

| **Methods** | **Theory** | **Research results** | **Advantage** | **Disadvantage** |
| --- | --- | --- | --- | --- |
| Membrane filtration and density gradient centrifugation | Use size differences, size and deformability | The cells were deformed at a flow rate of 2000 μL/min, and the capture rate was 95% [1] | Simple operation, not affected by surface markers | Low purity, low specificity and low sensitivity |
| Electrophoresis enrichment method | Capture of CTCs-cluster by physical methods using cell electrical characteristics | The CTCs-cluster is captured efficiently and the integrity of the cell cluster is maintained. Breast cancer (1 clusters/2 mL); Melanoma (0.3 clusters/2 mL); Prostate cancer (0.56 clusters/2 mL) [2] | High purity, high survival rate and repeatability | Low capture efficiency |
| Acoustic cell sorting | Acoustic sensors are placed and CTCs are sorted by ultrasonic wave | The clinical separation effect is good; The sorting speed was 7.5 mL/h, and the capture rate was > 86% [3] | No need for ligands, biological compatibility | Flux is low |
| Aptamer capture method | Using the negative sorting method of CTC-ICHIP chip | High sensitivity for further analysis of cell characteristics and subsequent studies.  The number of CTCs was 6.4/2 mL [4] | High repeatability, long shelf life, mild conditions, high affinity | The high cost |
| Immunomagnetic bead method, immobilized antibody microchannel | Positive sorting method using fishbone microfluidic sorting platform | With high capture efficiency, CTCs can be analyzed in situ for single cell isolation. The number of CTCs was 2.5-4/2 mL [5] | High specificity and sensitivity | Low efficiency, poor repeatability, high cost |
| DLD technology, inertial focusing technology, eddy current capture technology | Hydromechanics | The flow rate was 100-300 μL/min, and the tumor cell activity was high (89%-91%), which could be used for subsequent detection [6] | High flux, high purity, no biological markers, not easy to block | Low accuracy |

**References**

1. Liu Z, Huang F, Du J, Shu W, Feng H, Xu X,et al. Rapid isolation of cancer cells using microfluidic deterministic lateral displacement structure. Biomicrofluidics. 2013;7(1):11801.
2. Sarioglu AF, Aceto N, Kojic N, Donaldson MC, Zeinali M, Hamza B, et al. A microfluidic device for label-free, physical capture of circulating tumor cell clusters. Nat Methods. 2015;12(7):685-91.
3. Ding X, Peng Z, Lin SC, Geri M, Li S, Li P, et al. Cell separation using tilted-angle standing surface acoustic waves. Proc Natl Acad Sci USA. 2014;111(36):12992-7.
4. Ozkumur E, Shah AM, Ciciliano JC, Emmink BL, Miyamoto DT, Brachtel E, et al. Inertial focusing for tumor antigen-dependent and -independent sorting of rare circulating tumor cells. Sci Transl Med. 2013;5(179):179ra47.
5. Brinkmann F, Hirtz M, Haller A, Gorges TM, Vellekoop MJ, Riethdorf S, et al. A versatile microarray platform for capturing rare cells. Sci Rep. 2015;5:15342.
6. Park JS, Song SH, Jung HI. Continuous focusing of microparticles using inertial lift force and vorticity via multi-orifice microfluidic channels. Lab Chip. 2009;9(7):939-48.
